# Perceived restorativeness and environment quality in relation to well-being, residential satisfaction, and sense of community: an analysis in Northeast Italy

**DOI:** 10.3389/fpsyg.2025.1522098

**Published:** 2025-02-18

**Authors:** Laura Miola, Francesca Pazzaglia

**Affiliations:** ^1^Department of General Psychology, University of Padova, Padua, Italy; ^2^Inter-university Research Centre in Environmental Psychology (CIRPA), Rome, Italy

**Keywords:** residential satisfaction, well-being, sense of community, restorativeness, environmental quality

## Abstract

**Introduction:**

Residential satisfaction consists of pleasure derived from living in a place according to one’s needs, expectations, and outcomes. The present study examines the role of sociodemographic variables, perceived residential quality indicators, and restorativeness in predicting i) well-being, ii) residential satisfaction, and iii) sense of communities in northeast Italy.

**Methods:**

A total of 100 residents (47 women) in various cities in northeast Italy and 211 (112 women) residents in Piazzola sul Brenta (PD) took part in two studies. They answered demographic questions about self-reported restorativeness, residential environment quality, residential satisfaction, mental well-being, and sense of community.

**Results:**

After accounting for age, gender, and income, the results showed that perceived restorativeness enhances sense of community in the Northeast and Piazzola sul Brenta samples and predicts psychological well-being and residential satisfaction in Piazzola sul Brenta. Architectural and functional aspects contribute, respectively, to residential satisfaction and sense of community in both samples, and functional factors predict residential satisfaction for the Northeast sample. Place attachment plays a positive role in residential satisfaction and sense of community in the Northeast and Piazzola sul Brenta.

**Discussion:**

The study reveals a link between perceived restorativeness and residential satisfaction and well-being, providing insight for professionals and policy to improve urban quality.

## 1 Introduction

Urbanization consists of a process that changes rural areas into urban settlements, altering, in addition, the spatial distribution of a population from rural to urban areas. A total of 55.3% of the world’s population currently resides in urban areas, a percentage expected to rise to 60% by 2030 (United Nations, 2018). Doubtless, life in the cities offers unique social, cultural, and professional opportunities, but it also exposes inhabitants to environmental stressors, such as noise, crowding, traffic, and air pollution (Bilotta et al., 2019). Such environmental stressors can have a negative effect for citizens of all ages regarding their health through alterations of the inflammatory, immune and cardiovascular system, and at a psychological level (for example deterioration of cognitive processes and psychological well-being) (e.g., Zhang, 2018; Bilotta et al., 2019).

Rapid urban growth and its associated environmental stressors can negatively affect residential satisfaction and sense of community, with impacts that extend beyond health and psychological well-being. For example, a study revealed that 50% of residents experienced annoyance due to at least one environmental factor, such as noise, vibrations, odors, or light, with local traffic emerging as the most frequent source of disturbance. This study further demonstrated that residential dissatisfaction and diminished place attachment mediated the relation between environmental annoyance and a reduced quality of life (Pedersen, 2015).

Residential satisfaction consists of the pleasure derived from living and dwelling in a particular place according to one’s needs, expectations, and outcomes (Amérigo, 2002). Residential satisfaction is a multidimensional construct generally assessed by satisfaction with dwelling, neighborhood, neighbors, house and building (Adriaanse, 2007; Lu, 1999; Bonaiuto and Fornara, 2004). Various indicators have been identified to explain and predict residential satisfaction, including objective and subjective indicators of urban quality (e.g., Amérigo, 2002). Residential satisfaction indeed depends on the assessments of the physical characteristics of the urban quality, the affective bond with the place, and the activities and uses that are conducted in the place of residence (Bonaiuto et al., 2015; Bonaiuto and Fornara, 2004).

Related concept to residential satisfaction is sense of community considered a social-psychological factor expressing the connection between the inhabitants and their neighborhood or community environment. It can be defined as a sense of belonging among members with shared confidence that their needs will be fulfilled (McMillan and Chavis, 1986). Some research has shown that residential satisfaction and sense of community are positively associated with well-being and health (Aragonés et al., 2017; Chen et al., 2015; Stewart and Townley, 2020). Various environmental factors have been proposed to explain residential satisfaction and sense of community, including physical, social, functional, and affective factors (Bonaiuto and Fornara, 2004). Bonaiuto et al. (2015) created a comprehensive and reliable questionnaire to assess environmental quality indicators (Perceived Residential Environment Quality Indicators, PREQI) and validated its factorial structure (Fornara et al., 2010; Mao et al., 2015). The questionnaire identifies various dimensions: physical (e.g., architectural and urban planning), functional (e.g., services), socio-relational (e.g., neighbors), and contextual aspects (e.g., maintenance and cure), and comprises four items on place attachment. Place attachment refers to the emotional and affective significance that individuals associate with their home, which can also extend to their neighborhood, city, and region (Giuliani, 2003). Research has shown that strong place attachment bonds are positively associated with improved quality of life (Harris et al., 1995). Moreover, place attachment is often viewed as the emotional and affective antecedent of residential satisfaction (Bonaiuto et al., 2015).

Environmental restorativeness is another factor to consider in relation to residential satisfaction and well-being in cities (Bornioli and Subiza-Pérez, 2023). An environment can be defined as restorative if can facilitate the recovery and improve well-being and health in individuals (Hartig et al., 2014). Increasing evidence indicates that exposure to restorative environments can influence people’s cognitive, psychological, psychophysiological, and social resources in individuals at all ages (e.g., Berto, 2014; Hartig et al., 2014; Chen et al., 2016). Two main theories have been proposed to explain why people can benefit from exposure to certain types of environments: the attention restoration theory (Kaplan, 1995; Kaplan and Kaplan, 1989) and the stress recovery theory (SRT; Ulrich, 1983; Ulrich, 2023). According to the former, the environment fosters feelings of fascination (spontaneously attracting our interest and attention), being away (giving us the feeling of escaping from everyday routine and changing of experience), compatibility (congruence between the environmental features and people’s needs, goals, and inclinations), and extent (the coherence between the environmental elements and scope), with positive effects on people’s voluntary attention (Kaplan and Kaplan, 1989). Based on the SRT, Ulrich (1983) proposed that the interactions with restorative environments can provide physiological benefits, resulting in decreased stress and increased relaxation and positive mood (Ulrich et al., 1991).

The environments that have been extensively studied for their restorative properties are the natural ones, and it is well known that the exposure to real and virtual environments with natural elements can enhance people’s well-being and people’ resources (Berto, 2005; Lee et al., 2022). However, restorative characteristics can also belong to other types of environments, such as built and urban ones (Kaplan and Kaplan, 1989). Nevertheless, compared to the extensive body of research on natural environments, the literature on built environments is smaller. Urban places and urban environments, in the first stage were used as a control condition to study the effects of natural places (e.g., Ulrich et al., 1991), later several studies investigated urban places (such as squares, school campuses or urban parks) with the presence of greenery to clarify their restorative characteristics (e.g., Carrus et al., 2017; Sella et al., 2023; Rapuano et al., 2022; Staats, 2012; Weber and Trojan, 2018). Finally, a smaller number of studies have been conducted to investigate the restorative effects of natural as opposed to historical environments in laboratory settings, with participants viewing videos or images, suggesting that restorative characteristics may exist in historical environments (e.g., Reece et al., 2022). Only a few studies did researchers explore the restorative properties in real cities with historical heritage (e.g., Masullo et al., 2021; Scopelliti et al., 2019), suggesting the restorative values of historical centers, but their relationship with domains of well-being and residential satisfaction has not been investigated.

The Italian northeastern region stands out as a key area for the Italian economy (respectively, 12, 14, and 20% of Italian people, gross national product, and export), characterized by increasing urbanization (Istat, 2021); strong historical, social, cultural territorial backgrounds; and a variety of vocations, from industry to tourism and cultural heritage. Therefore, the residential satisfaction and psychological well-being of inhabitants in northeastern Italy are contrasted between two aspects: urbanization and an important historical and architectural heritage. The present study’s novelty consists of investigating whether and to what extent there is a link between perceived restorativeness and perceived residential environment quality with people’s residential satisfaction, sense of community, and well-being in cities of the Italian northeast. Understanding which aspects are related to residential satisfaction, sense of community, and psychological well-being can help maximize the benefits of high levels of urbanization while minimizing environmental degradation and other potential negative impacts of increasing numbers of city dwellers.

The present work is part of the Spoke 4 (“City, Architecture and Sustainable Design,” focusing on the interaction between individuals and environments) of the iNEST project, financially supported by the Italian National Recovery and Resilience Plan (PNRR Program). The iNEST project aims to extend the beneficial effects of digitalization and progress to northeastern Italy (Friuli-Venezia Giulia, Veneto, and the Autonomous Province of Trento and Bolzano).

In this theoretical and organizational framework, we aim to increase psychological knowledge of residential satisfaction, sense of community, and well-being, exploring the role of restorative experiences with the idea that the greater a place’s restorative value, the greater its inhabitants’ residential satisfaction and well-being experienced.

With these aims, we conducted two studies in the North-East of Italy. Study 1 involved a sample of inhabitants from several Italian cities in the northeastern regions of Veneto, Friuli Venezia-Giulia, and Trentino Alto-Adige. Study 2 allowed us to replicate the relationships among the variables found in Study 1 and test them in a circumscribed area, a small municipality in the province of Padua: Piazzola sul Brenta, that lies near the banks of the river it is named after. Piazzola sul Brenta was chosen following an in-depth analysis of its geographical, historical, and architectonical characteristics (see Montanari et al., 2023). It is a small town and therefore ensures that respondents live in a circumscribed area whose features and characteristics are better defined than in larger cities in the northeast of Italy, where neighborhoods and areas can be very different from each other. Moreover, Piazzola sul Brenta, compared to other cities in the Northeast, has well-defined boundaries and it is an exemplary blend of historical, artistic, and natural elements. At its center lies an ancient villa of architectural value (Villa Contarini), complemented by other historical features, such as the colonnade, all surrounded by a park. The historical and architectural analysis of the area, including the overlay of cartographic maps, highlighted that the emergence of the current urban layout was strongly influenced by the positioning of the villa and its park, suggesting that the historical feature (Villa Contarini) and the annexed natural elements (the villa’s parks) even today significantly affect the urban structure and, consequently, the lives of the inhabitants. A recent study by Miola et al. (2024) revealed that residents of Piazzola sul Brenta recognize the historical and economic significance of Villa Contarini and its park. Furthermore, higher levels of satisfaction and sense of community were associated with stronger negative emotions when imagining the loss of such places of historical and economic value.

The present article will primarily aim to explore which factors of environmental quality and restorativeness could predict well-being, residential satisfaction and sense of community in the in the North-East of Italy. Building on the data collected from the sample provided by Miola et al. (2024), the present studies expanded upon the work by analyzing the perceived quality factor and incorporating an additional sample of residents from the northeast region. On the basis of previous evidence (Bonaiuto and Fornara, 2004; Bonaiuto et al., 2015; Permentier et al., 2011), we expected that individual/social (e.g., age, income, place attachment) and environmental components (physical, functional, socio-relational, and contextual aspects) could predict residential satisfaction as well as sense of community and well-being. We expected that restorativeness could predict well-being (Ulrich et al., 1991) but could also positively predict residential satisfaction and sense of community in the Northeast of Italy. In particular, we expected that restorativeness would predict outcomes especially in Piazzola sul Brenta being a town with both natural (park) and historical (Villa) elements.

## 2 Study 1: the northeast of Italy

### 2.1 Participants

A total of 110 residents in northeast of Italy participated in the study. Ten participants were excluded because they lived in cities outside the northeast region. The final sample comprised 100 participants (47 women) who resided in various cities in northeast Italy (e.g., Venice, Vicenza, Padua, Trento, Bolzano, Trieste) from 20 to 65 years old (mean age = 31.14, SD = 9.61). Participants were recruited on the PROLIFIC platform. The Ethical Committee for Psychological Research at University of Padua approved the study (N° 215-a). All participants were informed about the purposes of the study before it was conducted and gave their informed consent, in accordance with the Declaration of Helsinki (World Medical Association, 2013).

### 2.2 Materials

General residential satisfaction (Bonaiuto et al., 2015; Fornara et al., 2007). Three questions assessing general inhabitants’ residential satisfaction on a 7-point Likert scale (1 = not at all to 7 = completely). The three items are (1) “Overall, how satisfied do you feel with living in this city?” (2) “Would you recommend this city to friends or acquaintances who are looking for housing?” and (3) “Do you plan to continue living in this city for a long time?”

The Warwick-Edinburgh Mental Wellbeing Scale (WEMWBS; Gremigni and Stewart-Brown, 2011; Tennant et al., 2007). The questionnaire consists of 12 items on a 5-point Likert scale (1 = strongly disagree to 5 = strongly agree) assessing mental well-being of respondents thinking about the last 2 weeks. A sample item is “I’ve been dealing with problems well.”

The Multidimensional Territorial Sense of Community scale (MTSCS; Prezza et al., 2009). The questionnaire consists of 19 items on a 5-point Likert scale (1 = strongly disagree to 5 = strongly agree) to measure territorial sense of community and sense of belonging. A sample item is “I feel I belong to the city community.”

Perceived Restorativeness Scale—Brief Version (PRS-11; Pasini et al., 2014). The questionnaire consists of 11 items on a 10-point Likert scale (0 = not at all to 10 = very much) to investigate the restorativeness of a place through its beneficial properties. It measures the following factors: fascination, being away, coherence, and scope. An example for fascination is “Places like this make me curious.” An example for “being away” is “Places like this are a refuge from daily worries.” An example for “coherence” is “There is a clear order in the physical arrangement of places like this.” Finally, an example for “scope” is “In places like this, there are few restrictions on my ability to move.” The subject is asked to rate his or her degree of agreement with statements.

The Perceived Residential Environment Quality Indicators (PREQIs; Bonaiuto et al., 2015). The questionnaire consists of 66 items on a 7-point Likert scale (0 = completely disagree to 6 = completely agree) to subjectively assess environmental urban quality. It comprises 4 main subscales regarding environmental qualities: (1) architectural and town-planning spaces (building aesthetics, density, volume and internal practicability); (2) social features (population, type of relations, sociability and cordiality, discretion and civility), (3) functional features (available services like schools, social care, sport, commercial and transport and social-cultural activities), and (4) contextual features (lifestyle, pollution, maintenance and care) and (5) neighborhood attachment. Sample items are: “The houses in the neighborhood are too attached to each other,” “There are green spaces in the neighborhood where you can relax,” “Vandalism often occurs in this neighborhood,” “The district is well provided for school services” and “Overall, this neighborhood is not polluted.”

### 2.3 Procedure

Participants read and completed the informed consent, then the demographic questions and questionnaires. Concerning the order of completion, the informed consent and demographic questionnaire were filled out at the beginning, followed by the PREQIs, PRS-11; WEMWBS; and MTSCS, which were presented in a randomized order. This study is part of a larger project (iNEST) in which other variables were also measured.

### 2.4 Statistical analysis

All analyses were conducted using RStudio. First, we assembled the descriptive statistics and reliability measures, including all the questionnaires. Then, linear models were computed to estimate the effects of environmental indicators and restorativeness on the dependent variables of (1) psychological well-being, (2) residential satisfaction, and (3) sense of community. The predictors were age, income, architectural, social, functional, contextual factors, place attachment, and restorativeness. AIC-based stepwise model selection was performed to select the best-fitting model and to diminish overfitting and favoring a more parsimonious model (Burnham et al., 2011). Each variable was standardized and added one at time. It was maintained in subsequent models when it lowered the AIC (Akaike information criterion; Wagenmakers and Farrell, 2004) and BIC by at least 2 units. For all the dependent variables, eight models were compared (see Supplementary material). Multicollinearity on each model was examined using the variance inflation factor (VIF), which ranged from 1.03 to 2.22, indicating low-moderate collinearity.

### 2.5 Results

#### 2.5.1 Northeast Italy sample

Table 1 shows the descriptive statistics and reliability. See Supplemental material for details of the linear models (stepwise AIC procedure).

**Table 1:** Descriptive statistics of the sample of the northeast of Italy and reliability.

	*M*	*SD*	Cronbach alpha	Max score
1. Residential satisfaction	14.34	4.46	0.89	21
2. Psychological well-being (WEMWBS)	41	8.43	0.91	60
3. Sense of community (MTSCS)	59.76	15.55	0.87	95
4.Restorativeness	64.21	15.39	0.89	121
5. PREQIs _Architecture	82.31	11.11	0.82	133
6. PREQIs _Social	38.78	6.83	0.76	63
7. PREQIs _Functional	77.65	13.61	0.87	140
8. PREQIs _Context	53.67	8.08	0.75	98
9. PREQIs _Attachment	13.96	5.38	0.91	24

For psychological well-being, the model that most improved the AIC index (best AIC = 265.63) included age, architectural, contextual factors, and restorativeness, accounting for 23% of the variance calculated using the adjusted R^2^ [*F*_(6, 93)_ = 6.86, *p* < 0.001]. No statistically significant predictors emerged.

For residential satisfaction, the model that most improved the AIC index (best AIC = 188.69) included age, income, architectural, social, functional factors, and attachment, accounting for 64% of the variance calculated using the adjusted R^2^ [*F*_(6, 93)_ = 31.1, *p* < 0.001]. A main effect of architectural (*B* = 0.15, CI [0.01, 0.30], *p* = 0.03) and functional (*B* = 0.18, CI [0.03, 0.33], *p* = 0.01) factors and place attachment (*B* = 0.61, CI [0.45, 0.77], *p* < 0.001) was found.

For sense of community, the model that most improved the AIC index (best AIC = 197.98) included age, income, and restorativeness, accounting for the 60% of the variance calculated using the adjusted R^2^ [*F*_(8, 91)_ = 23.6, *p* < 0.001]. A main effect of functional factor (*B* = 0.18, CI [0.03, 0.34], *p* = 0.02), place attachment (*B* = 0.49, CI [0.30, 0.67], *p* < 0.001), and restorativeness (*B* = 0.22, CI [0.05, 0.39], *p* = 0.01) emerged.

### 2.6 Discussion

The results of Study 1 offer new suggestions on one hand and confirms the outcomes of past studies on residential satisfaction (Bonaiuto et al., 1999, 2015) on the other. Regarding residential satisfaction and sense of community, a significant percentage of variance (around 60%) is explained by the variables of interest. In particular, functional aspects of the place of residence (e.g., welfare, recreational, commercial, and transport services) predict residential satisfaction and sense of community. Residential satisfaction is also predicted by architectural characteristics: neighborhoods where buildings are perceived as less dense, more pleasant, and well planned are more likely to be associated with higher residential satisfaction with dwellings. The considered variables explained a smaller percentage of variance (23%) in well-being. This was expected because well-being depends on many factors (e.g., physical health, socioeconomic status, and life conditions; Link and Phelan, 1995; McKay et al., 2020) that were not considered in this study, whose focus was environmental factors. However, it is still noteworthy that factors related to the environment’s physical dimensions can indeed explain a moderate percentage of variance in perceived well-being. Finally, the variable that was of greatest interest in the study (perceived restorativeness) explains a significant proportion of the sense of community experienced by people living in a territory. Sense of community is a relevant construct because it is the basis of social life. From our study, we can conclude that it is associated with functional factors (services), affective and emotional factor (place attachment), and the degree of perceived restorativeness.

## 3 Study 2: Piazzola sul Brenta

Study 1, even if interesting and novel in its results, suffered from the limitation of surveying people living in cities whose size, population density, and level of urbanization differed. In Study 2, we tested our model in a specific town (Piazzola sul Brenta) located in the northeast of Italy whose geographical, architectonic, and historical characteristics were thoroughly analyzed in a previous study (Montanari et al., 2023). We were interested in verifying whether the same variables as those found in Study 1 could explain well-being, residential satisfaction, and sense of community in this context. We also sought to test whether in a small center characterized by historical and natural peculiar elements, perceived restorativeness may have a crucial role.

### 3.1 Participants

A sample of 211 Residents in Piazzola sul Brenta (112 women) from 18 to 77 years old (mean age = 40.66, SD = 15.05) took part in the study. Participants were recruited through social media and word of mouth. The Ethical Committee for Psychological Research at University of Padua approved the study (N° 215-a). All participants were informed about the purposes of the study before it was conducted and gave their informed consent in accordance with the Declaration of Helsinki (World Medical Association, 2013). The sample used in this study was previously analyzed in Miola et al. (2024), where additional variables were examined as part of the broader PNRR project, albeit with a different focus.

### 3.2 Materials

Materials, procedure, and statistical analysis are the same as in the previous study.

### 3.3 Results

For psychological well-being, the stepwise AIC procedure suggested that the model that most improved the AIC index (best AIC = 566.96) included age, income, architectural, social, functional, context, and restorativeness. The hypothesized predictors accounted for 19% of the variance calculated using the adjusted R^2^ [*F*_(6, 204)_ = 8.44, *p* < 0.001]. A statistically significant main effect of restorativeness (*B* = 0.22, CI [0.07, 0.37], *p* = 0.004) emerged (Table 2).

**Table 2:** Descriptive statistics of the sample of Piazzola sul Brenta and reliability.

	*M*	*SD*	Cronbach alpha
1. Residential satisfaction	17.07	4.94	0.86
2. Psychological well-being (WEMWBS)	43.86	7.19	0.87
3. Sense of community (MTSCS)	67.07	14.28	0.88
4.Restorativeness	63.78	19.55	0.92
5. PREQIs _Architecture	85.37	12.59	0.87
6. PREQIs _Social	36.33	6.48	0.78
7. PREQIs _Functional	75.81	15.38	0.89
8. PREQIs _Context	52.18	9.95	0.83
9. PREQIs _Attachment	17.01	5.40	0.87

For residential satisfaction, the stepwise AIC procedure suggested that the model that most improved the AIC index (best AIC = 396.60) included age, architectural, social, functional, contextual attachment, and restorativeness. The hypothesized predictors accounted for 63% of the variance calculated using the adjusted R^2^ [*F*_(8, 202)_ = 52.95, *p* < 0.001]. A statistically significant main effect of age (*B* = 0.01, CI [0.01, 0.02], *p* < 0.001), architectural factor (*B* = 0.12, CI [0.01, 0.23], *p* = 0.02), place attachment (*B* = 0.49, CI [0.39, 0.60], *p* < 0.001), and restorativeness (*B* = 0.25, CI [0.14, 0.36], *p* < 0.001) emerged.

For sense of community, the stepwise AIC procedure suggested that the model that most improved the AIC index (best AIC = 433) included age, architectural, social, functional, contextual, attachment, and restorativeness. The hypothesized predictors accounted for the 60% of the variance calculated using the adjusted R^2^ [*F*_(7, 203)_ = 39.96, *p* < 0.001]. A statistically significant main effect of functional factor (*B* = 0.30, CI [0.16, 0.43], *p* < 0.001), place attachment (*B* = 0.30, CI [0.18, 0.41], *p* < 0.001), and restorativeness (*B* = 0.23, CI [0.11, 0.35], *p* < 0.001) emerged.

### 3.4 Discussion

Study 2 substantially confirm the results of Study 1, indicating environmental properties are important in explaining well-being, residential satisfaction, and sense of community. Similarly, as found in Study 1, architectural and functional elements predict residential satisfaction and sense of community. Interestingly, perceived restorativeness predicts all the considered variables, assuming a crucial role for all the considered outcomes. Finally, unlike in the general sample, in Piazzola sul Brenta, age explains residential satisfaction, with older people expressing higher satisfaction.

## 4 General discussion

In the present study, we investigate psychological well-being, residential satisfaction, and sense of community and their relationship with perceived residential environment quality and restorativeness in the cities of the northeast region of Italy. The general aim of the study stems from the iNEST project, within the National Recovery and Resilience Plan.

More specifically, the main aim of the study is to investigate the effects of individual factors, perceived residential environment quality, and the role of restorativeness on psychological well-being, residential satisfaction, and sense of community in cities of the northeast, with a focus on a small city in the province of Padua, Piazzola sul Brenta. For these aims, we proposed an online survey that inhabitants of various cities in the northeast of Italy (e.g., Venice, Vicenza, Padua, Trento, Bolzano, Trieste) completed, and a second sample included residents of Piazzola sul Brenta (PD).

The results showed that restorativeness is associated with psychological well-being for residents of Piazzola sul Brenta, suggesting that the more people perceived their cities as restorative, fascinating, coherent, and capable of breaking out of the ordinary, the more they referred to their psychological well-being. Interestingly, none of the other indicators of perceived residential quality emerged to affect people’s psychological well-being on Piazzola sul Brenta, highlighting the importance of perceived restorativeness. Therefore, the presence and the quality of restorative environments can provide benefits for their inhabitants’ psychological well-being.

Restorativeness also predicted residential satisfaction and sense of community in Piazzola sul Brenta.

Consequently, restorativeness also plays a role in fostering not only a personal and individual well-being but also a more environmental dimension, such as residential satisfaction and sense of community. A possible explanation is that restorative environments promote and provide space for social interactions and consist of an opportunity that the town offers, thus contributing to the sense of belonging and community.

Expanding upon the work of Miola et al. (2024) to include inhabitants of the regional area, the northeastern sample reveals that restorativeness appears to play a role in fostering sense of community but does not significantly influence well-being or residential satisfaction. This suggests that in larger northeastern cities, this construct is likely shaped by other factors. Conversely, overall, restorativeness emerged as a more prominent factor for the residents of Piazzola sul Brenta, where it became a significant predictor of both well-being and residential satisfaction. These findings align with the hypothesis that in places featuring a strong interplay between historical and natural elements, such as Piazzola sul Brenta, environmental restorativeness assumes a greater importance compared to larger cities, where such features may be less interconnected and present. As for the indicators of perceived residential environment quality, the results showed that architectural and functional factors emerged as important indicators of residential satisfaction for the Northeast sample, suggesting that the more architectural elements of the cities are perceived as pleasant and well organized in terms of density, esthetics, and volumetry, the higher is the residential satisfaction perceived. Moreover, the more the services (functional aspect) are perceived as adequate and satisfactory, the higher is the residential satisfaction. The role of functional aspects does not emerge for residential satisfaction in Piazzola sul Brenta suggesting how in large cities this may be a more relevant aspect. Functional factors however contributed to sense of community in the cities in the northeast and Piazzola sul Brenta, suggesting that the quality of the town’s services improve sense of community and belonging.

Finally, attachment to a place contributes to residential satisfaction and sense of community in the northeast cities and Piazzola sul Brenta. These findings suggest that the more participants felt attachment and an affective relationship with the city, the more they perceived residential satisfaction. This result makes it clear that environments are places with not only physical connotations but also affective meanings. The affective bond and emotion aroused in people by the place in which they live contributes to their satisfaction and feeling of belonging there.

Finally, only for inhabitants of Piazzola sul Brenta, age emerges as a significant predictor, highlighting that as people grow older, their residential satisfaction in Piazzola sul Brenta increases. This result may suggest that older adults living in Piazzola sul Brenta may be more satisfied with their homes and cities than younger people, leading to increased residential satisfaction (Lu, 1999; Chapman and Lombard, 2006). Another possible explanation associated with this result could be that as people age, their attachment to places increases (Mandal, 2016), thus contributing to better residential satisfaction. No other individual factor seems to have an association with the outcomes, and this result was unexpected. Income, in particular, was expected to be a significant predictor, especially in relation to residential satisfaction in both the Northeast region and in Piazzola sul Brenta.

It is important to acknowledge the current study’s limitations. First, it should be noted that the findings are correlational and rely on an Italian sample. Therefore, further investigation should include other countries to generalize the results to other contexts and cultures. Another limitation might be that it did not include objective measures of urban quality indicators to better understand the role of objective variables in comparison to subjective ones. Moreover, the number of subjects and statistical power that could be improved and expanded in the future. Finally, a limitation of the study lies in the strong correlation observed between the sense of community and place attachment subscale, which may indicate potential overlap between these constructs. This could have implications for the interpretation of their distinct contributions to the outcomes examined. To conclude, the results provide evidence that restorativeness plays a dual role: not only does it contribute to individual psychological well-being, but it also has a broader impact on people’s satisfaction with their living environment and their sense of community. This expands the theoretical understanding of environmental restorativeness, emphasizing its significance beyond individual outcomes to encompass communal and residential dimensions. However, this effect is particularly pronounced in residential areas where historical and natural elements are interconnected and interrelated, aligning with theoretical expectations.

Place attachment also emerged as a relevant factor across both northeastern cities and Piazzola sul Brenta, reinforcing previous findings on its importance (Bonaiuto et al., 2015). Interestingly, income, which was expected to influence residential satisfaction, did not appear significant for either sample. Conversely, age emerged as a factor influencing residential satisfaction in Piazzola sul Brenta, suggesting that as people age, their satisfaction increases in locations characterized by the coexistence of historical, artistic, and natural features.

Therefore, a promising strategy to improve psychological well-being and residential satisfaction in cities is to improve the number and the quality of restorative environments. In fact, restorative environments, in addition to restoring people’s psychological and psychophysiological resources and contributing to urban well-being, can provide opportunities for socialization, which contributes to a sense of belonging and community in cities. In addition, this study showed that the important urban quality factors contributing to residential satisfaction for the northeast are functional and architectural factors above all. Therefore, municipalities should especially work on these aspects to improve citizens’ well-being and satisfaction. Finally, the results also show the importance of emotional aspects in residential satisfaction and sense of community. In particular, the higher the satisfaction, the greater the attachment to a place.

## Data Availability

The raw data supporting the conclusions of this article will be made available by the authors, without undue reservation.
