# Supplementary material for: Perceived restorativeness and environment quality in relation to well-being, residential satisfaction, and sense of community: an analysis in Northeast Italy
Source: Front Psychol. 2025 Feb 18;16:1522098. doi: 10.3389/fpsyg.2025.1522098 (PMC11876131; doi:10.3389/fpsyg.2025.1522098)

Supplementary Material

***Table S1-S6. Linear models for the Northeast sample***

**Table S1**

| Psychological wellbeing (Northeast sample) | | | |
| --- | --- | --- | --- |
| Model | Variable added | AIC | BIC |
| m0 | + Age | 284.97 | 292.78 |
| m1 | + Gender | 288.70 | 301.73 |
| m2 | + Income | 284.95 | 295.37 |
| m3 | + Architectural factor | **276.56** | 286.98 |
| m4 | + Social factor | 275.45 | 288.48 |
| m5 | + Functional factor | **272.53** | 289.15 |
| m6 | + **Contextual factor** | **267.56** | 283.19 |
| m7 | + Attachment | 267.30 | 285.54 |
| **m8** | **+** Restorativeness | **265.63** | **283.26** |

**Table S2**

| Residential satisfaction (Northeast sample) | | | |
| --- | --- | --- | --- |
| Model | Variable added | AIC | BIC |
| m0 | + Age | 281.17 | 288.99 |
| m1 | + Gender | 284.73 | 297.76 |
| m2 | + Income | **279.36** | 289.78 |
| m3 | **+ Architectural factor** | **256.06** | 269.08 |
| m4 | + Social factor | **251.38** | 267.02 |
| m5 | + **Functional factor** | **233.58** | 252.07 |
| m6 | + Contextual factor | 234.58 | 255.42 |
| **m7** | **+ Attachment** | **188.69** | **209.53** |
| m8 | + Restorativeness | 188.94 | 212.39 |

**Table S3**

| Sense of community (Northeast sample) | | | |
| --- | --- | --- | --- |
| Model | Variable added | AIC | BIC |
| m0 | + Age | 286.31 | 294.13 |
| m1 | + Gender | 286.90 | 299.92 |
| m2 | + Income | **282.48** | 292.90 |
| m3 | + Architectural factor | **272.63** | 285.66 |
| m4 | + Social factor | **258.66** | 274.30 |
| m5 | **+ Functional factor** | **239.39** | **257.62** |
| m6 | + Contextual factor | 240.19 | 261.03 |
| m7 | **+ Attachment** | **202.99** | 223.84 |
| **m8** | **+ Restorativeness** | **197.98** | **221.42** |

**Table S4**

| Psychological wellbeing (Northeast sample) | | | |  |  |
| --- | --- | --- | --- | --- | --- |
|  | Preditors | B | CI | St. Error | *p* |
|  | + Age | 0.01 | [-0.01- 0.03] | 0.00 | 0.19 |
|  | + Architectural factor | 0.02 | [-0.19 - 0.24] | 0.11 | 0.82 |
|  | + Functional factor | 0.12 | [-0.07 - 0.33] | 0.10 | 0.21 |
|  | + Contextual factor | 0.22 | [0.00 - 0.44] | 0.11 | 0.05 |
|  | + Restorativeness | 0.22 | [-0.00 - 0.45] | 0.11 | 0.06 |

**Table S5**

| Residential satisfaction (Northeast sample) | | | |  |  |
| --- | --- | --- | --- | --- | --- |
|  | Preditors | B | CI | St. Error | *p* |
|  | + Age | 0.01 | [-0.00 - 0.02] | 1.66 | 0.10 |
|  | + Income | 0.004 | [-0.06 - 0.07] | 0.13 | 0.90 |
|  | **+ Architectural factor** | **0.15** | **[0.01 - 0.30]** | **2.19** | **0.03** |
|  | + Social factor | -0.04 | [-0.20 - 0.11] | -0.58 | 0.56 |
|  | **+ Functional factor** | **0.18** | **[0.03 - 0.33]** | **2.51** | **0.01** |
|  | **+Place attachment** | **0.61** | **[0.45 - 0.77]** | **7.48** | **<.001** |

**Table S6**

| Sense of community (Northeast sample) | | | |  |  |
| --- | --- | --- | --- | --- | --- |
|  | Preditors | B | CI | St. Error | *p* |
|  | + Age | -0.00 | [-0.01 - 0.01] | 0.00 | 0.92 |
|  | + Income | -0.01 | [-0.08 - 0.06] | 0.03 | 0.80 |
|  | + Architectural factor | -0.13 | [-0.29 - 0.03] | 0.08 | 0.11 |
|  | + Social factor | 0.15 | [-0.01 - 0.32] | 0.08 | 0.07 |
|  | **+ Functional factor** | **0.18** | **[0.03- 0.34]** | **0.07** | **0.02** |
|  | **+ Place attachment** | **0.49** | **[0.30 - 0.67]** | **0.09** | **<.001** |
|  | **+ Restorativeness** | **0.22** | **[0.05 - 0.39]** | **0.08** | **0.01** |

***Table S7-S12. Linear models for the Piazzola sul Brenta sample***

**Table S7**

| Psychological wellbeing (Piazzola sul Brenta sample) | | | |
| --- | --- | --- | --- |
| Model | Variable added | AIC | BIC |
| m0 | + Age | 603.69 | 613.74 |
| m1 | + Gender | 608.19 | 628.30 |
| m2 | + Income | 604.23 | 617.63 |
| m3 | + Architectural factor | **591.57** | 604.98 |
| m4 | + Social factor | **589.68** | 606.44 |
| m5 | + Functional factor | **582.18** | 602.28 |
| m6 | + Contextual factor | **573.39** | 596.86 |
| m7 | + Attachment | 575.39 | 602.21 |
| **m8** | **+ Restorativeness** | **566.96** | **593.77** |

**Table S8**

| Residential satisfaction (Piazzola sul Brenta sample) | | | |
| --- | --- | --- | --- |
| Model | Variable added | AIC | BIC |
| m0 | **+ Age** | 591.87 | 601.92 |
| m1 | + Gender | 597.40 | 617.51 |
| m2 | + Income | 593.77 | 607.18 |
| m3 | **+ Architectural factor** | **558.47** | 571.88 |
| m4 | + Social factor | **556.67** | 573.43 |
| m5 | + Functional factor | **533.74** | 553.85 |
| m6 | + Contextual factor | **519.55** | 543.01 |
| m7 | **+ Attachment** | **415.95** | 442.76 |
| **m8** | **+ Restorativeness** | **396.60** | **426.76** |

**Table S9**

| Sense of community (Piazzola sul Brenta sample) | | | |
| --- | --- | --- | --- |
| Model | Variable added | AIC | BIC |
| m0 | + Age | 603.34 | 613.39 |
| m1 | + Gender | 605.15 | 625.26 |
| m2 | + Income | 605.14 | 618.54 |
| m3 | + Architectural factor | **567.32** | 580.73 |
| m4 | + Social factor | **563.11** | 579.87 |
| m5 | **+ Functional factor** | **508.56** | **528.67** |
| m6 | + Contextual factor | **488.94** | 512.40 |
| m7 | **+ Attachment** | **445.91** | 472.73 |
| **m8** | **+ Restorativeness** | **433.00** | **463.16** |

**Table S10**

| Psychological wellbeing (Piazzola sul Brenta sample) | | | |  |  |
| --- | --- | --- | --- | --- | --- |
|  | Preditors | B | CI | St. Error | *p* |
|  | + Age | 0.004 | [-0.004- 0.01] | 0.004 | 0.32 |
|  | + Architectural factor | 0.01 | [-0.14 - 0.17] | 0.08 | 0.81 |
|  | + Social factor | 0.08 | [-0.05 - 0.22] | 0.07 | 0.25 |
|  | + Functional factor | 0.07 | [-0.10 - 0.25] | 0.09 | 0.43 |
|  | + Contextual factor | 0.17 | [-0.00 - 0.35] | 0.09 | 0.05 |
|  | **+ Restorativeness** | **0.22** | **[0.07 - 0.37]** | **0.07** | 0.004 |

**Table S11**

| Residential satisfaction (Piazzola sul Brenta sample) | | | |  |  |
| --- | --- | --- | --- | --- | --- |
|  | Preditors | B | CI | St. Error | *p* |
|  | **+ Age** | **0.01** | **[0.01 - 0.02]** | **0.002** | **<.001** |
|  | **+ Architectural factor** | **0.12** | **[0.01 - 0.23]** | **0.05** | **0.02** |
|  | + Social factor | 0.004 | [-0.08 - 0.09] | 0.04 | 0.92 |
|  | + Functional factor | 0.10 | [-0.02 - 0.22] | 0.06 | 0.10 |
|  | + Contextual factor | -0.02 | [-0.14 - 0.09] | 0.06 | 0.67 |
|  | **+Place attachment** | **0.49** | **[0.39 - 0.60]** | **0.05** | **<.001** |
|  | **+Restorativeness** | **0.25** | **[0.14 - 0.36]** | **0.05** | **<.001** |

**Table S12**

| Sense of community (Piazzola sul Brenta sample) | | | |  |  |
| --- | --- | --- | --- | --- | --- |
|  | Preditors | B | CI | St. Error | *p* |
|  | + Age | 0.002 | [-0.003 - 0.008] | 0.003 | 0.46 |
|  | + Architectural factor | 0.02 | [-0.08 - 0.14] | 0.05 | 0.62 |
|  | + Social factor | 0.02 | [-0.08 - 0.12] | 0.05 | 0.68 |
|  | **+ Functional factor** | **0.30** | **[0.16 - 0.43]** | **0.06** | **<.001** |
|  | + Contextual factor | 0.09 | [-0.04 - 0.22] | 0.06 | 0.17 |
|  | **+Place attachment** | **0.30** | **[0.18 - 0.41]** | **0.05** | **<.001** |
|  | **+ Restorativeness** | **0.23** | **[0.11 - 0.35]** | **0.06** | **<.001** |

**Figure 1, 2 and 3. Histograms for outcomes**

**Figure 1**


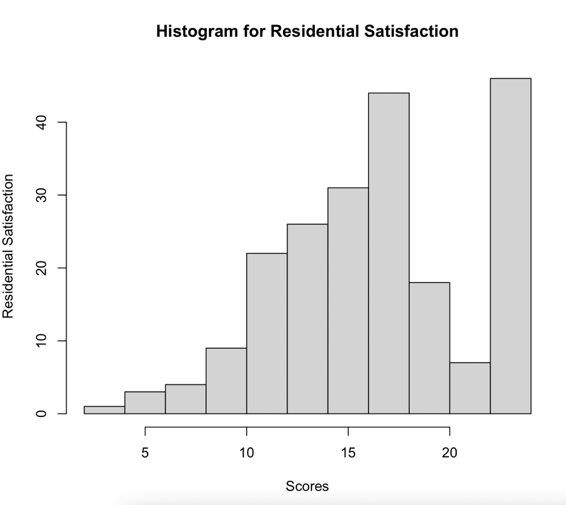


**Figure 2**


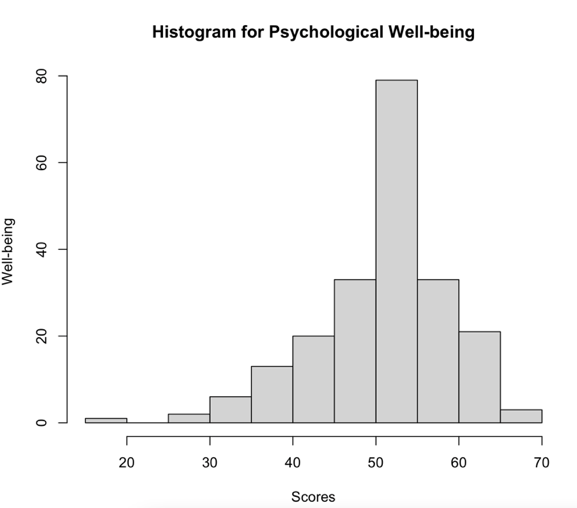


**Figure 3**


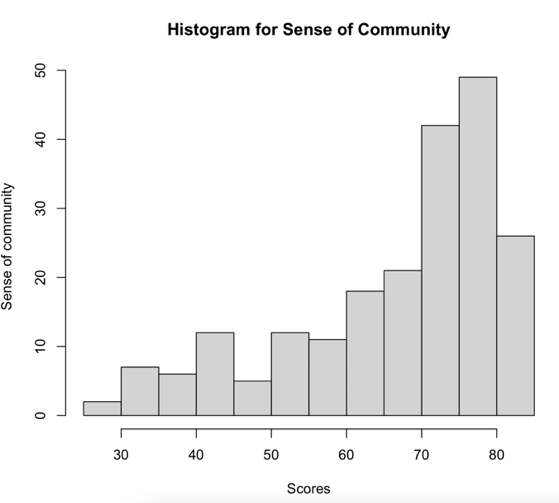

Supplement: Supplementary file 1 [file Data_Sheet_1.docx]
